# Coronectomy as a surgical approach to impacted mandibular third molars: a systematic review

**DOI:** 10.1186/s13005-015-0068-7

**Published:** 2015-04-10

**Authors:** Andrea Martin, Giuseppe Perinetti, Fulvia Costantinides, Michele Maglione

**Affiliations:** Unit of Oral Surgery, School of Dental Sciences, University of Trieste, Trieste, Italy

**Keywords:** Coronectomy, Inferior alveolar nerve, Review

## Abstract

The aim of this systematic review was to evaluate the clinical effectiveness of the surgical technique of coronectomy for third molars extraction in close proximity with the inferior alveolar nerve.

A literature survey carried out through PubMed, SCOPUS and the Cochrane Library from inceptions to the last access in January 31, 2014, was performed to intercept randomised clinical trials, controlled clinical trials, prospective cohort studies or retrospective studies (with or without control group) that examined the clinical outcomes after coronectomy. The following variable were evaluated: inferior alveolar nerve injury, lingual nerve injury, postoperative adverse effects, pulp disease, root migration and rate of reoperation. Ten articles qualified for the final analysis. The successful coronectomies varied from a minimum of 61.7% to a maximum of 100%. Coronectomy was associated with a low incidence of complications in terms of inferior alveolar nerve injury (0%-9.5%), lingual nerve injury (0%-2%), postoperative pain (1.1%-41.9%) and swelling (4.6%), dry socket infection (2%-12%), infection rate (1%-9.5%) and pulp disease (0.9%). Migration of the retained roots seems to be a frequent occurrence (2%-85.3%).

Coronectomy appears to be a safe procedure at least in the short term, with a reduced incidence of postoperative complications. Therefore, a coronectomy can be indicated for teeth that are very close to the inferior alveolar nerve. If a second operation is needed for the remnant roots, they can be removed with a low risk of paresthesia, because the roots are generally receded from the mandubular nerve.

## Introduction

Extraction of an impacted mandibular third molar has the potential risk of causing temporary or permanent neurologic disturbances of the inferior alveolar nerve (IAN) [1]. The incidence of IAN injury (IANI) reported in the literature ranges from 1.3% to 5.3% [2-6]. The risk of this complication depends mainly on the position of the impacted tooth in relation to the inferior alveolar canal before surgery [3]. If there is close proximity between the IAN and the roots, the incidence may be as high as 19% [7].

Injury to the IAN can occur from compression of the nerve, either indirectly by forces transmitted by the root during elevation or directly by elevators. The nerve may also become transected by rotary instruments or during removal of a tooth whose root is grooved or perforated by the IAN. Several researches have tried to correlate radiographic markers to the relationship between the IAN and the root of the tooth [8-10]. These radiographic signs only indicate to surgeons that there is an increased risk of nerve damage associated with the removal of the corresponding wisdom tooth, but they cannot help prevent the nerve deficit if the tooth is bound to be removed.

After a clear indication for extraction is defined, surgical removal of an impacted third molar with the roots in close contact with the IAN should attempt to minimize the risk of irreversible neurological complications.

Several approaches in this regard have been proposed. Checchi et al. [11] and Alessandri Bonetti et al. [12] introduced orthodontic-assisted extraction of impacted third molars, which has also been adopted by others [13]. This technique can improved periodontal healing distal to the second molar, but it also can be time consuming and costly.

Coronectomy has also been presented in the literature as a way to reduce neurological complications. This alternative surgical procedure was first proposed in 1984 and has continued to be studied [7,14-25].

The method aims to remove only the crown of an impacted mandibular third molar while leaving the root undisturbed, thereby avoiding direct or indirect damage to the IAN. Fissure burs are used to reduce the remaining roots to be at least 3 mm below the crest of the lingual and buccal plates. The pulp is left untouched and the root is checked for any mobility. Thereafter the wound is thoroughly debrided and irrigated with saline and finally closed primarily with sutures [14].

It is known that broken fragments of vital teeth generally heal without complications; if a root is broken during extraction of a regular uninfected tooth, it can safely be left *in situ* [15,26]. Coronectomy exploits this assumption. But not all third molar teeth are suitable for coronectomy. Teeth with acute infection and mobile teeth should be excluded, because root remnants of those teeth may act like foreign bodies. In addition, teeth that are horizontally impacted along the course of the inferior alveolar canal may be unsuitable, because sectioning of a tooth could endanger the nerve [14]. When making a decision about a coronectomy, it is necessary to determine the correct relationship between the root apices and the inferior alveolar canal. At this point, different radiological imaging techniques can be used. Dental computerized tomography can give very precise informations about the root-canal relationship, likely being the best choice for this purpose.

Until January 31, 2014, only a systematic review on the topic was available, which however, considered only 4 case–control studies [27]. Thus, the objective of the present systematic review was to further elucidate the clinical outcomes of the coronectomy when there is a high risk of neurological damage to the IAN extrapolating the results also from not still reviewed studies that compare this surgical technique with the total extraction of impacted mandibular third molars.

## Methods

Systematic reviews synthesize the evidence from scientific studies to provide informative answers to scientific questions by including a comprehensive summary of the available evidence [18].

### Search strategy and study selection

This systematic review follows the PRISMA statements and did not use- any previous systematic reviews as a template [28]. Herein, all of the studies that examined the clinical outcomes after coronectomy technique through a literature survey carried out through PubMed (www.ncbi.nlm.nih.gov/pubmed), SCOPUS (www.scopus.com) and the Cochrane Library (www.thecochranelibrary.com) from inceptions to the last access in January 31, 2014. “(Coronectomy) OR (Odontectomy)” was used as search algorithm. No language restrictions were used. Finally, a manual search was also performed by scoring the references within the studies examined. The studies retrieved had to be randomised clinical trials (RCTs), controlled clinical trials (CCTs) and prospective cohort studies (PCSs) or retrospective studies (RSs) with or without control group. Case series, case reports, studies enrolling less than 10 subjects, comments, expert opinion, letters to the Editor, reviews, studies that analysed the same sample of a pre-existing study were excluded.

Studies in which patients had high risk of IANI, as revealed by radiography (panoramic radiography or Cone Beam CT) were included. Specifically, the criteria for high risk of nerve injury included: displacement of the inferior alveolar canal by the roots; narrowing of the inferior alveolar canal; periapical radiolucent area; narrowing of third molar roots; darkening of third molar roots; curving of third molar roots; interruption and loss of lamina dura of nerve canal.

Studies that adopted coronectomy as surgical treatment or compare coronectomy with total removal of lower third molar extractions with high risk of IANI were also collected. Studies in which lower third molars had any of the followings were included: pericoronitis, periodontal disease of the second mandibular molar, follicular or any clinical condition that does not affect the vitality of the tooth such as caries, endodontic diseases, apical pathology, apical cystic or neoplastic lesions. Studies in which patients presented any of the followings were excluded: clinical signs of systemic infection; medically compromised conditions because of diabetes, chemotherapy, previous radiotherapy, immunologic disease, bone disease (osteoporosis, osteosclerosis or osteopetrosis); existing neural disorders; craniofacial syndromes with pre-existing IAN deficit; any plans for orthognathic surgery; pregnancy; patients younger than 16 years old (premature roots).

In addition studies in which follow-up of the clinical outcomes after surgery was greater than two months were only collected. Inclusion and exclusion criteria for articles selection were as follows:

Inclusion criteria:

- Randomized clinical trials (RCTs); controlled clinical trials (CCTs); and prospective cohort studies (PCSs) or retrospective studies (RSs) with or without control group;
- Patients who had high risk of inferior alveolar nerve injury (IANI), as revealed by radiography (OPT or CBCT); specifically, the criteria for high risk of nerve injury included: displacement of the inferior alveolar canal by the roots; narrowing of the inferior alveolar canal; periapical radiolucent area; narrowing of third molar roots; darkening of third molar roots; curving of third molar roots; interruption and loss of lamina dura of nerve canal;
- Study that adopted coronectomy as surgical treatment or compare coronectomy with total removal for lower third molar extractions with high risk of nerve injury were included. Specifically, as described by Pogrel et al. [14] coronectomy is a technique of removing the crown of a tooth but leaving a part of the roots untouched. The crown of a tooth is removed and fissure burs are used to reduce the remaining roots to be at least 3 mm below the crest of the lingual and buccal plates. The pulp was left untouched and the root was checked for any mobility. After the wound was thoroughly debrided and irrigated with saline and finally was closed primarily with sutures. In contrast, total removal is the conventional surgical extraction technique which removes a tooth completely;
- Patients were included if their lower third molars had any of the following: pericoronitis, periodontal disease of the second mandibular molar, follicular or any clinical condition that doesn’t affect the vitality of the tooth;
- Studies in which follow-up of the clinical outcomes after surgery was greater than two months.

Exclusion criteria:

- Case report, case series, study enrolling less than 10 subjects, comments, expert opinion, letters to the Editor, reviews, studies that analysed the same sample of a pre-existing study;
- Patients were excluded if they had any of the following: clinical signs of systemic infection; medically compromised conditions because of diabetes, chemotherapy, previous radiotherapy, immunologic disease, bone disease (osteoporosis, osteosclerosis or osteopetrosis); existing neural disorders; craniofacial syndromes with pre-existing IAN deficit; any plans for orthognathic surgery; pregnancy; patients younger than 16 years old (premature roots);
- Patients were excluded if their lower third molars had any of the following: non-vital third molars; caries; endodontic disease; wisdom teeth associated with apical pathology or apical cystic or neoplastic lesions.

### Data items

The following data items were collected: study design (RCTs, CCTs, PCs or RSs with or without control group); sample size; age and sex distribution of the sample; number of teeth involved; adjunct pharmacological treatments (behavioural indications and possible drug therapy); surgical failure, defined when the removal of the crown caused mobilization of the roots leading to complete tooth extraction with subsequent risk of injury to the IAN (the decision to remove the mobile roots was based on the premise that they had lost their vitality and therefore had an increased susceptibility to infection) [7]; inferior alveolar nerve injury (IANI), divided in transitory (t-IANI) or permanent (p-IANI); lingual nerve injury (LNI), divided in transitory (t-LNI) or permanent (p-LNI); postoperative adverse effects (pain assessment (P), defined as cases reported to be painful one week postoperatively; swelling assessment (SW), defined as cutaneous swelling of the operated side; dry socket infection (DSI), defined as the presence of severe pain, loss of the blood clot in the socket, and wound breakdown; infection rate (I), defined as the presence of pus, fever and pain); pulp disease, defined as the presence of pulpitis or appearance of periapical radiolucency in postoperative radiograph evaluation; migration of the roots away from IAN after the surgical treatment (measured as the radiographic distance from the point of interception of the upper white line of the inferior dental canal and long axis of root, to the apex of the root along the long axis); reoperation (need for a second surgery to remove the retained roots in case of root exposure, infection or pulp disease); follow-up; clinical implications according to the authors.

### Assessment of study quality and risk of bias in individual or across studies

Evaluation of methodological quality of published studies gives an indication of the strength of evidence provided. However, no single approach in assessing methodological soundness may be appropriate to all systematic reviews. Therefore contextual, pragmatic, and methodological considerations are followed when assessing study quality. Herein, a modified quality evaluation method from Antczak et al. [29] was used that followed pre-established characteristics, along with the systematic scores that were assigned to the individual retrieved articles:

- Study design: 3 points: RCT, 2 points: CCT, 1 points: PCS, RS;
- Adequacy in sample selection description based on four criteria: (i) sample size; (ii) age and sex; (iii) systemic health conditions: 2 points: full description, 1 points: partial description;
- Adequacy in preoperative radiographic evaluation of relationship between tooth and IAN: 2 points: preoperative radiographic evaluation performed with CBCT, 1 points: preoperative radiographic evaluation performed with OPT;
- Adequacy in treatment description based on three criteria: (i) description of surgical technique; (ii) description of surgical team; (iii) description of adjunct pharmacological treatments (behavioural indications and possible drug therapy): 2 points: full description, 1 points: partial description;
- Adequacy in development of an accurate follow-up: 2 points: full description, 1 points: partial description;
- Adequacy in description of clinical outcomes and number of findings: (i) inferior alveolar nerve injury (IANI) or lingual nerve injury (LNI); (ii) pain assessment; (iii) dry socket infection; (iv) infection rate; (v) pulp disease; (vi) root migration; (vii) reoperation. 3 points: full description (≥6 outcomes), 2 points: partial description (3 to 5 outcomes), 1 points: poor description (≤2 outcomes);
- Adequacy of statistical analysis: 2 points: inferential statistics, 1 points: descriptive statistics
- Prior estimation of sample size: 1 point.

The quality of the studies, was considered as follows: low (total score ≤11 points), medium (total score ≥12 and ≤13 points), medium/high (total score ≥14 and ≤15 points), high: total score ≥16 points.

## Results

**Figure 1:**
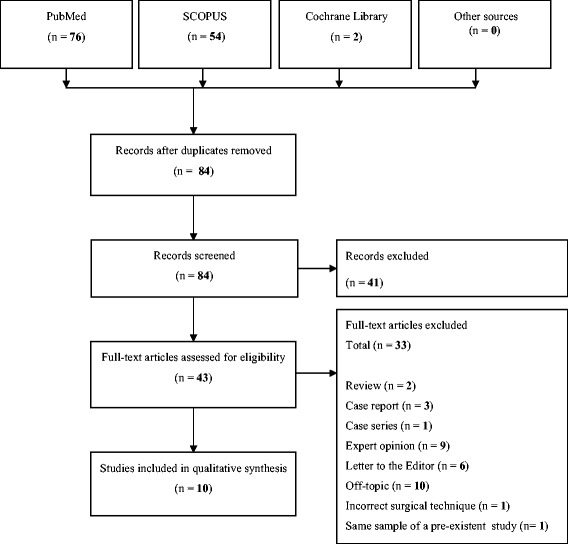
**Flow diagram of the search strategy.**

The results of the automatic and manual searches are shown in Figure 1. According to the automatic search, a total of 84 articles were retrieved.

Thirty-three of the 43 full-text articles assessed for eligibility were excluded for the followings:

- Review (n = 2) [27,30];
- Case report (n = 3) [18,31,32];
- Case series (n = 1) [19];
- Expert opinion (n = 9) [33-41];
- Letter to the editor (n = 6) [42-47];
- Off-topic (n = 10) [23,48-56];
- Incorrect surgical technique (n = 1) [57];
- Same sample of a pre-existent study (n = 1) [58].

Ten studies were judged to be relevant to the present study according to the inclusion/exclusion criteria [7,14,16,17,22,24,25,59-61]. Finally, in the manual search, no more relevant studies were included. The full details of the studies included are summarised in Table 1.

**Table 1:** **Summarized data of the 10 studies included in the review**

**Study**	**Study design**	**Sample size (n)**	**Sex and age (range or mean or mean ± SD)**	**Teeth (n)**	**Adjunct pharmacological treatments**	**Surgical success or failure [n(%)]**	**IANI [n(%)]**	**LNI [n(%)]**	**Adverse effects [n(%)]**	**Pulp disease [n(%)]**	**Root migration [n(%)] and/or (mm and timepoint)**	**Reoperation [n(%)]**	**Follow-up (range or mean ± SD)**	**Clinical implications**
*O’Riordan et al.* [22]	RS	52	31 yrs*	95	Antibiotics	S: 87 (91.6%)	t-IANI: 3 (3.4%)	t-LNI: 1 (1.1%)	I: 3 (3.4%)	No	NA	3 (3.4%)	24 months*	Effective alternative to extraction
						F: 8 (8.4%)								
*Progrel et al.* [14]	PCS	41	NA	50	Preoperative antibiotics	S: 50 (100%)	No	t-LNI: 1 (2%)	NA	NA	15 (30%) 2–3 mm in 6 months	3 (6%)	22 months	Effective alternative to extraction
						F: 0 (0%)								
*Renton et al.* [7]	RCT	128	M 30; F 64	94	Preoperative Chlorhexidine mouth washes	S: 58 (61.7%)	No	No	P: 8 (13.8%)	No	5 (13.2%) 2 mm in 12 months	No	25 ± 13 months	Low risk of complications than extraction
									I: 3 (5.2%)					
									DSI: 7 (12%)					
			28.2 ± 5.9 yrs*			F: 36 (38.3%)	t-IANI: 3 (8%)	No	P: 4 (11.1%)	/	/	/		
									DSI: 4 (11,1%)					
*Dolanmaz et al.* [25]	PCS	43	M 23; F 20	47	Antibiotics	S: 46 (97.9%)	p-IANI: 1 (2.2%)	NA	NA	No	3.4 mm in 6 months	No	9.3 months	Effective alternative to extraction
			18–38 yrs			F: 1 (2.1%)					3.8 mm in 12 months			
											4 mm in 24 months			
*Hatano et al.* [16]	CCT	107*	M 27; F 75	107*	NA	S: 102 (95,3%)	t-IANI: 1 (1%)	No	P: 19 (18.6%)	NA	87 (85.3%)	5 (4.9%)	13.5 ± 14.8 months	Low risk of complications than extraction
			32.4 ± 10.4 yrs			F: 0 (0%)*			DSI: 2 (2%)					
									I: 1 (1%)					
*Leung and Cheung* [17]	RCT	171	M 70; F 101	171	Paracetamol and codein for 3 days	S: 155 (90.6%)	t-IANI: 1 (0.6%)	No	P: 65 (41.9%)	No	96 (62.2%) 1.9 mm in 3 months	1 (0.6%)	10.6 ± 7.7 months	Low risk of complications than extraction
									I: 9 (5.8%)		36 (23.6%) 3 mm in 6 months			
											3 (2%) 3.1 mm in 24 months*			
			27.2 ± 7.3 yrs			F: 16 (9.4%)	t-IANI: 1 (6.2%)	No	NA	/	/	/	11.4 ± 7.9 months	
*Cilasun et al.* [24]	CCT	88	27.2 yrs	88	Antibiotics Benzydamine HCL plus Chlorhexidine gluconate for 5 days	S: 86 (97.7%)	No	NA	P: 1 (1.1%)	No	NA	1 (1.2%)	17 months	Effective alternative to extraction
									I: 1 (1.1%)					
						F: 2 (2.3%)	No	NA	No	/	/	/		
*Goto et al.* [61]	PCS	101	M 79; F 37	116	NA	S: 116 (100%)	No	No	DSI: 7 (6%)	1 (0.9%)	3 mm in 12 months	8 (6.9%)	12 months	Safety technique in 12 months
			33 yrs*			F: 0 (0%)								
*Monaco et al.* [59]	PCS	37	M 17; F 20	43	Antibiotics for 4 days Chlorhexidine for 10 days Ibuprofen	S: 43 (100%)	No	No	P: 1 (2%)	NA	1.6 mm in 3 months	1 (2.3%)	12 months	Effective alternative to extraction
			31 ± 2 yrs			F: 0 (0%)			SW: 2 (4.6%)		2 mm in 6 months			
									DSI: 1 (2%)		2 mm in 12 months			
*Patel et al.* [60]	RS	21	M 10; F 11	21	Antibiotics	S: 20 (95.2%)	t-IANI: 2 (9.5%)	NA	I: 2 (9.5%)	NA	9 (43%)	1 (5%)	2-40 months	Effective alternative to extraction
			41.3 yrs			F: 0 (0%)	p-IANI: 1 (4.8%)		DSI: 1 (4.8%)					

**CCT**: controlled clinical trial; **DSI**: dry socket infection; **F**: female (in age and sex column) or failed coronectomy (in surgical success or failure column); **I**: infection rate; **p-IANI**: permanent inferior alveolar nerve injury; **t-IANI**: transient inferior alveolar nerve injury; **p-LNI**: permanent lingual nerve injury; **t-LNI**: transient lingual nerve injury; **M**: male; **NA**: not available; **P**: pain; **PCS**: prospective cohort study; **RCT**: randomized clinical trial; **RS**: retrospective study; **S**: success coronectomy; **SW**: swelling; **SD**: standard deviation; **/**: not applicable; *: value derived from graphs or text of the study.

### Studies design and population

The 10 studies included comprised 2 RCTs [7,17], 2 CCTs [16,24], 4 PCSs [14,25,59,61] and 2 RSs [22,60].

The sample size of patients undergoing corononectomy ranged from a minimum of 21 subjects [60] to a maximum of 171 subjects [17] and the mean age was included between 27.2 years [17] and 41.3 years [60]. Only four studies considered a control group [7,16,17,24]. Seven studies monitored both sexes [16,17,25,59-61], while three studies did not report this information [14,22,24]. Only the study of Pogrel et al. has not provided any information both as regards age and sex [14]. In all of the studies, the subjects were systemically healthy.

### Teeth investigated, surgical treatment and adjunct pharmacological treatments

The coronectomies were performed on a sample of teeth that ranged from a minimum of 21 teeth [60] to a maximum of 171 teeth [17].

Only a few studies have specified the indications and postoperative medical therapy provided to patients. In six studies [14,22,24,25,59,60] an antibiotic therapy was prescribed postoperatively and two of these [24,59] have also used chlorhexidine mouth rinses. One study prescribed chlorhexidine mouth rinses preoperatively [7] and two studies made also prophylactic antibiotic therapy [14,59]. Two studies have prescribed anti-inflammatory therapy postoperatively [17,59]. Two studies did not provide any postoperative indications [16,6].

### Failure of the coronectomy

The criteria used to measure the failure of the therapy were clearly defined in six studies [7,16,17,24,25,59]. In five of these, an unsuccessfull coronectomy was assessed if there was complete extraction of the tooth due to the roots mobilization during the decrowning procedure (surgical criterium) [7,17,24,25,59]. In one study the authors defined as “failed” coronectomy the procedure that lead to extraction of the remnant roots due to infection [16]. Irrespective of the criteria used to assess failure (when reported), the failed coronectomies ranged from 0% [14,59,61] to 38.3% [7].

### Clinical outcomes

Five studies [14,24,59,61] reported t-IANI that ranged in percentage from a minimum of 0% [59] to a maximum of 9.5% [60], and two studies [25,60] have reported p-IANI that ranged from 2.2% [25] to 4.8% [60]. Renton et al. found nineteen IANI (19%) in the control group (n = 102), none after successful coronectomy (n = 58), and three (8%) after failed coronectomy (n = 36) (p = 0.01) [7]. In the study of Hatano et al., six patients of the control group (n = 118) showed signs of IANI (5%) [16]. Of these, three were diagnosed with p-IANI (2.5%). In the coronectomy group, one patient (1%) complained t-IANI. The difference between groups was not statistically significant (p = 0.126). Leung and Cheung found postoperative IANI for nine teeth in the control group (5.10%, 9/178), whereas only one case occurred after coronectomy (0.6%, 1/155) [17]. The difference was statistically significant (p = 0.023). Six patients in the control group developed a t-IANI, whereas the remaining three (33.3%) had a p-IANI. The authors also found one case of t-IANI in the sixteen failed coronectomies (6.2%). In the study by Cilasun et al., two cases of t-IANI (2.8%) were observed in the control group (87 teeth) while no patients of the study group (88 teeth) developed IANI (0%) [24]. Two studies [14,22] reported t-LNI that varied in percentage from 1.1% [22] to 2% [14]. There was no evidence of a p-LNI. No LNI was observed both in the control and coronectomy groups [7,16,17]. Three studies did not evaluate this outcome [24,25,60].

Five studies [7,16,17,59,61] have found clinical pain (P) after the surgical treatment that ranged in percentage from 1.1% [24] to 41.9% [17]. Renton et al. found P in 22 patients (21.6%) of the control group (n = 102) in respect with 8 patients (13.8%) of the coronectomy group and 4 patients (11.1%) of the failed coronectomy group [7]. The difference was not statistically significant. Leung and Cheung found that among the control group, 57.3% (102/178) of teeth were reported to be painful 1 week postoperatively [17]. The corresponding proportion in the coronectomy group was 41.9% (65/155), which was statistically different (p = 0.005). However, there were no statistical differences between the two groups 1 to 24 months after surgery. Also, Hatano et al. found significant differences in post-operative P in the comparison between the control group (6.78%, 8/118) and the coronectomy group (18.6%, 19/102) with a p-value of 0.012 [16]. In the study by Cilasun et al., no cases (0%) of P were observed in the control group (n = 87), one case was observed after a total of 88 coronectomies (1.1%) [24]. One study reported postoperative SW in 4.6% of cases after success coronectomy [59]. Five studies [7,16,59-61], related postoperative dry socket infections (DSI) that ranged from a minimum of 2% [16] to a maximum of 11.1% [7]. Renton et al. found a similar incidence of DSI in the control group (9.6%, 10/102), coronectomy group (12%, 7/58), and failed coronectomy group (11.1%, 4/36) [7]. Conversely, Hatano et al. observed a higher percentage of DSI in the control group (8.5%, 10/118) in respect with coronectomy group (2%, 2/102) [16]. The difference was significant (p = 0.039). Also Leung and Cheung found a significant difference (p = 0.036) between the control group (2.8%, 5/178) and the coronectomy group (0%, 0/171) [17]. Cilasun et al. reported one case of DSI in the control group (1.1%) and no cases in the coronectomy group [24]. Five studies [16,17,22,24,60] have reported infection (I) from 1% [16] to 9.5% [60]. Results by Renton et al. showed one case of I in the control group (1%, 1/102), three cases in the coronectomy group (5,2%, 3/58) and no cases in the failed coronectomy group [7]. No statistical significance was observed. Hatano et al. found a percentage of I of 3.4% (4/188) in the control group and of 1% (1/102) in patients undergoing coronectomy (p = 0.376) [16]. Leung and Cheung found an I incidence rate of 6.7% (n = 178) in the control group and of 5.8% (n = 155) in the coronectomy group, with no statistical differences [17]. Cilasun et al. observed no cases of I in the control group (n = 87) and one case in the coronectomy group (1,1%, 1/88) [24]. Two studies did not take into account this value in their analysis of clinical outcomes [14,25]. One study reported a case of pulp disease whose value in percentage was 0.9% [61]. Four studies did not take into account this value in their analysis of clinical outcomes [14,16,59,60].

Five studies [7,14,16,17,60] reported the number of cases of root migration that ranged from a minimum of 2% [17] to a maximum of 85.3% [16]. Six studies have reported the distance in millimetres of the root migration in relation to the displacement time; not all of these studies have considered the same time-points [7,14,17,25,59,61]. The mean movement of the root remnants ranged from 1.6 mm [59] to 1.9 mm [17] in 3 months, from 2 mm [59] to 3.4 mm [25] in 6 months, from 2 mm [59] to 3.8 mm [25] in 12 months, and from 3.1 mm [17] to 4 mm [25] in 24 months. Two studies did not take into account this parameter in their analysis of clinical outcomes [22,24].

Eight studies [14,16,17,22,24,59-61] have required the need for reoperation of the retained that varied in percentage from a minimum of 0.6% [17] to a maximum of 6.9% of cases performed [61].

The mean follow-up ranged from a minimum of 9.3 months [25] to a maximum of 25 months [7]. One study expressed the value of follow-up as a range of 2–40 months [60].

Clinical outcomes are summarized in Table 1.

### Main reported results and clinical implications

In their conclusions, six studies have considered the coronectomy as a reliable procedure to be considered as an effective alternative to total removal in cases of close proximity between third molars and IAN [14,22,24,25,59,60]. Three studies affirmed that coronectomy is a surgical procedure that has lower risk of complications than total extraction of third molars [7,16,17]. One study concluded that coronectomy is a safe technique up to 12 months follow-up [61].

### Quality analysis and risk of bias in individual studies

The results of quality analysis are shown in Table 2. The quality was high in one study [17], medium/high in three studies [7,16,59], medium in two studies [24,61] and low in all of the other four studies [14,22,25,60].

**Table 2:** **Overview of the quality of included studies**

**Study**	**Study design**	**Sample description**	**Radiographic evaluation**	**Treatment description**	**Follow-up development**	**Clinical outcomes description**	**Adequacy of statistical analysis**	**Previous estimate of sample size**	**Quality score**	**Judged quality standard**
*O’Riordan et al.* [22]	RS	Partial	OPT	Partial	Full	Partial	Descriptive	No	9	Low
*Progrel et al.* [14]	PCS	Partial	OPT	Partial	Full	Poor	Descriptive	No	8	Low
*Renton et al.* [7]	RCT	Full	OPT	Full	Full	Full	Inferential	No	15	Medium/High
*Dolanmaz et al.* [25]	PCS	Full	OPT	Full	Full	Partial	Descriptive	No	11	Low
*Hatano et al.* [16]	CCT	Full	CBCT	Partial	Full	Full	Inferential	No	14	Medium/High
*Leung and Cheung* [17].	RCT	Full	OPT	Full	Full	Full	Inferential	Yes	16	High
*Cilasun et al.* [24]	CCT	Partial	CBCT	Full	Full	Full	Descriptive	No	13	Medium
*Goto et al.* [61]	PCS	Full	CBCT	Partial	Full	Partial	Inferential	No	12	Medium
*Monaco et al.* [59]	PCS	Full	CBCT	Full	Full	Full	Inferential	Yes	15	Medium/High
*Patel et al.* [60]	RS	Partial	CBCT	Full	Partial	Partial	Descriptive	No	10	Low

**CBCT**: Cone Beam Computed Tomography; **CCT**: controlled clinical trial; **OPT**: panoramic radiography; **PCS**: prospective cohort study; **RCT**: randomized clinical trial; **RS**: retrospective study.

The sample description was classified as adequate in six studies [7,16,17,25,59,61], and as not adequate in four studies [14,22,24,60]. All of the studies described the sample size, but only six studies extensively detailed age, sex and systemic health of the subjects included [7,16,17,25,59,61].

The preoperative radiographic evaluation of relationship between tooth and IAN was depth performed with CBCT only in five studies [16,24,59-61], while the other five studies used the OPT [7,14,17,22,25].

The treatment description was adequate in six [7,17,24,25,59,60], while it was not adequate in four studies for the absence of a surgical team and adjunct pharmacological treatments description [14,16,22,61].

The follow-up was correctly described and performed by nine studies [7,14,16,17,22,24,25,59,61]; the last one study was not accurate in development of the follow-up [60].

Clinical outcomes were adequately researched and described in five studies [7,16,17,24,59], while were partially described in four studies [22,25,60,61]. In the only study with a description classified as poor, the lack of information regarded pain assessment, dry socket, infection rate and pulp disease [14].

The statistical methods were judged appropriate in five studies that presented an inferential statistics [7,16,17,59,61]; in contrast, five studies reported only descriptive [14,22,24,25,60]. Finally, only two studies used *a priori* calculation of sample size [17,59].

## Discussion

Coronectomy was proposed as a clinical procedure more than 20 years ago, but has not been commonly performed, largely owing to the lack of well-designed evidence-based trials to support its use [39].

This systematic revision was conducted to evaluate the clinical effectiveness and reliability of the surgical technique of coronectomy in third molars extraction in close proximity with the IAN.

The surgical procedure described by Pogrel et al. used by the studies involved in this revision seems to be reliable and reproducible [14]. Appropriate sections on a CBCT can better show proper anatomical relationship between the roots and the IAN than panoramic radiographs, and display the positions of the roots and the IAN in 3-dimensional view.

Results indicate that coronectomy of wisdom teeth is a safe technique when the wisdom tooth shows radiographic signs of close proximity of the IAN to the root.

The rate of postoperative failure after coronectomy seems to be low, on average less than 10%; Renton et al. have detected a failure rate of 38.8% and considering the medium/high quality score this data are worth of consideration [7]. The t-IANI was found to be low in percentage with values less than 8%; Patel et al. [60] reported t-IANI in 9.5% of cases and only Dolanmaz et al. [25] and Patel et al. [60] reported p-IANI, even in 4.8% of cases. However, these studies did not specify if the p-IANI or t-IANI was found following a successful coronectomy or a failed coronectomy and subsequent total extraction; these studies having a low quality score and their results would have less relevance. The LNI was found to be extremely rare and only present as t-LNI maximum in 2% of cases in the study of Pogrel et al., a study with low quality score [14].

Adverse effects of coronectomy constitute a concern to clinicians. The presence of pain seems to be constant in 10-20% of cases after coronectomy, and reached very high rates, amounting to 41.9% in the study by Leung and Cheung, the only study with a high quality score and with a very large sample size [17].

The swelling is rare and was only detected in a study in 4.6% of cases [59]. Although a smaller proportion of patients experiencing postoperative DSI after coronectomy, this complication has reached 12% of cases in the study of Renton et al., a study with a medium/high quality score [7]. Although immediate postoperative infection can occur, it is very unusual, particularly if antibiotics and primary closure are used; Patel et al. have only reached values of 9.5%, but it is a low quality study [60].

Regarding the need for antibiotic therapy in the case of coronectomy, Pogrel et al. explained that all patients involved in the study were placed on prophylactic antibiotics preoperatively, because it is felt that antibiotics should be in the pulp chamber of the tooth at the time it is transacted [14]. This should improve the clinical post-operative outcomes. However, some studies have indicated that antibiotics were unnecessary [7,47].

Renton et al., Leung and Cheung and Hatano et al. showed the highest percentages of adverse effects and these studies showed the greatest quality score [7,16,17]. It is assumed that the non-administration of a postoperative antibiotic therapy has favoured the appearance of a greater number of complications. Conversely, the studies that have shown minor adverse effects were those in which antibiotic therapy was administered postoperatively. Furthermore, the study by Monaco et al. was the only that administered antibiotic prophylaxis before coronectomy and prolonged therapy postoperatively with a very low incidence of infection [59].

Only a case of pulp disease was detected by Goto et al., specifically a case of pulpitis, which led to reoperation and to residual root extraction [61].

According to the results of animal studies, it is not necessary to provide dental pulp treatment to the remaining root [62-64]. Sencimen et al. [23] reported that better prognostic results were obtained in patients who had not received root canal treatment with mineral trioxide aggregates after coronectomy [62,64-66].

Coronal root migration after coronectomy was a common finding. Leung and Cheung and Hatano et al. revealed that more than half of the roots migrated at high rate for 3–6 months postoperatively and then gradually stopped at 12 to 24 months [16,17].

All the studies that have evaluated the root migration have detected actual migration of the root and all studies suggested that most migratory component would be present in the first 6 months postoperatively, with an average migration of 2–3 mm. Goto et al. affirmed that factors that correlated significantly with root migration were age, sex and root morphology [61]. With regard to sex, the mean migration was significantly greater in female than male patients and also greater in younger patients. Migration by the conical roots was significantly greater than that by the enlarged or clubbed roots.

Root migration would be slowed down and gradually halted as the bone regenerates and remodels. However, residual root movement was unpredictable. Reoperation rate owing to postoperative root migration in oral cavity or infection ranged from 0.6% to 6.8%.

The mean follow-up of the studies was enough for the assessment of clinical outcomes such as IANI, LNI, P, SW, DSI, I and pulp disease but too short for a proper assessment of the root migration.

In agreement with Pogrel et al. it would be is not necessary to recall the patient after 6 months, unless he or she becomes symptomatic [14]. On the other hand, some asserts that a follow-up period of 25 months is required to evaluate the incidence of nerve injury, but not of late eruption, which can occur up to 10 years after the initial operation [21]. A longer follow-up period may therefore show which proportion of these retained roots will eventually erupt, cause a late infection or require removal [7].

The present results thus warrant further research with larger sample size and longer follow-up to fully describe the long-term outcome of the electively retained root. Furthermore, since different studies considered in this review did not describe the clinical protocol employed, further publications are encouraged to point out attention on operator technique that is a very important variable that could influence the final clinical success.

## Conclusions

Coronectomy appears to be a safe procedure at least in the short term, with a reduced incidence of postoperative complications and risk of IANI. The success requires both good patient and case selection and operator technique. Therefore, a coronectomy can be indicated for teeth that are very close to the IAN. If a second operation is needed for the remnant roots after a coronectomy, the roots can be removed with a low risk of paresthesia, because the roots would have receded from the inferior alveolar canal.

## Abbreviations

IAN: Inferior alveolar nerve
IANI: Inferior alveolar nerve injury
RCTs: Randomised clinical trials (RCTs)
CCTs: Controlled clinical trials
PCSs: Prospective cohort studies
RSs: Retrospective studies
t-IANI: Transitory inferior alveolar nerve injury
p-IANI: Permanent inferior alveolar nerve injury
LNI: Lingual nerve injury
t-LNI: Transitory lingual nerve injury
p-LNI: Permanent lingual nerve injury
P: Pain
SW: Swelling
DSI: Dry socket infection
I: Infection
CBCT: Cone beam CT
OPT: Panoramic radiography
